# Development of a Mammalian Cell Line for Stable Production of Anti-PD-1

**DOI:** 10.3390/antib13040082

**Published:** 2024-10-03

**Authors:** Erika Csató-Kovács, Pál Salamon, Szilvia Fikó-Lászlo, Krisztina Kovács, Alice Koka, Mónika András-Korodi, Emőke Antal, Emília Brumă, Brigitta Tőrsők, Szilárd Gudor, Ildikó Miklóssy, Kálmán Csongor Orbán, Csilla Albert, Emese Éva Bálint, Beáta Albert

**Affiliations:** 1 Department of Bioengineering, Faculty of Economics, Socio-Human Sciences and Engineering, Sapientia Hungarian University of Transylvania, 1 Libertatii Sq, 530104 Miercurea Ciuc, Romania; kovacserika@uni.sapientia.ro (E.C.-K.); salamonpal@uni.sapientia.ro (P.S.); kokaalice@uni.sapientia.ro (A.K.); gudorszilard@uni.sapientia.ro (S.G.); miklossyildiko@uni.sapientia.ro (I.M.); orbancsongor@uni.sapientia.ro (K.C.O.); albertcsilla@uni.sapientia.ro (C.A.); balintemese@uni.sapientia.ro (E.É.B.); 2 Corax-Bioner CEU S.A., 1 Miko Str., et. 1, Cam. 100, 530174 Miercurea Ciuc, Romania; korodimonika@uni.sapientia.ro (M.A.-K.); antalemoke@uni.sapientia.ro (E.A.); 3 Faculty of Natural Sciences, University of Pécs, 7 Ifjúság Útja St., 7624 Pécs, Hungary; laszloszilvia@uni.sapientia.ro (S.F.-L.); kovacskrisztina@uni.sapientia.ro (K.K.); bodor.emilia@yahoo.com (E.B.); torsokbrigi@yahoo.com (B.T.); 4 C. D. Nenițescu Institute of Organic Chemistry, 202B Splaiul Independenței, Sector 6, 060023 București, Romania

**Keywords:** immune checkpoint molecule, anti-PD-1, CHO DG44, Nivolumab, monoclonal antibody production

## Abstract

**Background/Objectives:** Immune checkpoint blockade, particularly targeting the programmed cell death 1 (PD-1) receptor, is a promising strategy in cancer immunotherapy. The interaction between PD-1 and its ligands, PD-L1 and PD-L2, is crucial in immune evasion by tumors. Blocking this interaction with monoclonal antibodies like Nivolumab can restore anti-tumor immunity. This study aims to develop a stable expression system for Nivolumab-based anti-PD-1 in the Chinese Hamster Ovary (CHO) DG44 cell line using two different expression vector systems with various signal sequences. **Methods:** The heavy chain (HC) and light chain (LC) of Nivolumab were cloned into two expression vectors, pOptiVEC and pcDNA3.3. Each vector was engineered with two distinct signal sequences, resulting in the creation of eight recombinant plasmids. These plasmids were co-transfected into CHO DG44 cells in different combinations, allowing for the assessment of stable antibody production. **Results:** Both pOptiVEC and pcDNA3.3 vectors were successful in stably integrating and expressing the Nivolumab-based anti-PD-1 antibody in CHO DG44 cells. This study found that the choice of signal sequence significantly influenced the quantity of antibodies produced. The optimization of production conditions further enhanced antibody yield, indicating the potential for large-scale production. **Conclusions:** This study demonstrates that both pOptiVEC and pcDNA3.3 expression systems are effective for the stable production of Nivolumab-based anti-PD-1 in CHO DG44 cells. Signal sequences play a critical role in determining the expression levels, and optimizing production conditions can further increase antibody yield, supporting future applications in cancer immunotherapy.

## 1. Introduction

A significant share of the demand for biologics today is represented by monoclonal antibodies, resulting in an increasing number of therapeutic monoclonal antibodies (mAbs) in various stages of development and an increasing search for innovative solutions for their production. In today’s competitive market, the most important criteria for mAbs development include maintaining the desired quality characteristics, shortening time to market, maintaining cost efficiency, and ensuring manufacturing flexibility [1]. Therapeutic mAbs are used in the treatment of various diseases such as cancer, inflammatory bowel disease (Crohn’s disease, ulcerative colitis), and rheumatoid arthritis [2]. The popularity of these antibodies is largely due to their high specificity, efficacy, molecular stability, and broad range of medical applications [2,3].

PD-1 is a type I transmembrane receptor consisting of 288 amino acids [4] and is expressed on the surface of T and B cells. Its role is to inhibit the immune response and establish self-tolerance by suppressing the inflammatory activity of T cells. While it protects the body from autoimmune diseases, it also diminishes the activity of previously activated tumor-reactive effector T cells through PD-L1/PD-L2 interactions, thus reducing their ability to destroy cancerous cells [5].

Nivolumab is a human immunoglobulin G4 (IgG4) monoclonal antibody, also known as Opdivo, and the first PD-1 immune checkpoint inhibitor approved for the treatment of advanced non-small cell lung cancer with and without squamous cell carcinoma after chemotherapy. Nivolumab binds to the receptor PD-1, inhibiting the receptor’s interaction with the ligands PD-L1 and PD-L2, which contributes to the development of an anti-tumor immune response [6]. The monoclonal antibody Nivolumab is used alone or in combination with other mAbs, such as ipilimumab (anti-CTLA4) in cancer immunotherapies [7]. In 2014, the anti-PD-1 monoclonal antibody, Nivolumab, was approved by the Food and Drug Administration (FDA) for patients with melanoma, followed by its approval for renal cell carcinoma in 2015 [8].

The most commonly used mammalian cell lines for therapeutic antibody production include mouse-derived NS0 and Sp2/0 cell lines, human-derived PER.C6 and HEK293 cell lines, and Chinese hamster ovary cell lines (CHOs). The most important consideration when selecting an expression system is to ensure high productivity with appropriate product quality characteristics [9]. CHO cell lines have several advantageous features: they can grow in suspension culture and serum-free, chemically defined media. Cultivation in suspension culture allows large-scale production in bioreactors, while the use of a chemically defined medium allows for optimization studies reproducibility between batches, with a better safety profile than media containing human- or animal-derived proteins [10].

One drawback of protein production in mammalian cells is low specific productivity, which can be compensated by gene amplification. Among the robust gene amplification systems used in CHO cells, dihydrofolate reductase (DHFR)- or glutamine synthetase (GS)-mediated amplification is fairly successful [11]. These systems are commonly utilized in the biopharmaceutical industry to achieve a high-level and stable expression of recombinant proteins [12]. An increase in target gene expression amplification based on the activation of the DHFR enzyme has been shown to enhance the expression of integrated genes [13]. The DHFR enzyme catalyzes the conversion of dihydrofolate (DHF) to tetrahydrofolate (THF), an essential precursor for nucleotide synthesis [14]. The inhibition or lack of DHFR activity leads to suppression of de novo nucleotide (thymidine and hypoxanthine) biosynthesis. The absence of these nucleotides causes the inhibition of RNA and DNA synthesis, DNA replication, and ultimately cell death [13]. The original CHO cell line was created by Dr. Puck in 1956 by isolating spontaneously immortalized fibroblasts from Chinese hamster ovarian cell culture [15]. The CHO DG44 cell line was developed by the elimination of the sequence encoding DHFR from the CHO genome [16]. Integration of the DHFR gene along with the gene of interest with an expression vector in the CHO DG44 cells enables the selection of a transfected cell population and the subsequent development of stable cell lines in media without exogenous nucleotides [12]. Methotrexate (MTX) blocks DHFR activity, inhibiting the conversion of DHF-THF, resulting in reduced nucleotide synthesis [14]. The addition of MTX not only improves the selection stringency but also induces the amplification of DHFR to develop resistance to MTX [12]. However, an active DHFR enzyme is necessary for cell survival, so the cell compensates for the inhibition of MTX by increasing the DHFR copy number together with the gene of interest [17]. This induced gene amplification strategy typically results in higher productivity of the gene of interest [13]. CHO cells are capable of efficient post-translational modifications, e.g., glycosylation, while the expressed recombinant proteins are compatible and bioactive within the human body [6].

Both monocistronic and bicistronic expression plasmids are used for the expression of antibody light and heavy chains [18]. In a two-monocistronic expression plasmid system, the expression level of each antibody chain can be influenced by transfection efficiency, genomic integration site, and gene copy number [19].

Polycistronic vectors used for antibody expression contain an internal ribosome entry site (IRES) or a furin/2A element, allowing for the expression of multiple transgenes from the same vector. The IRES facilitates the binding of the ribosomal complex to the vector [20]. These elements allow the expression of the heavy and light chains that make up the antibody from a single mRNA in a bicistronic (IRES) or monocistronic (Furin/2A) arrangement [21]. In the study by Mizuguchi et al., the expression level of the second gene mediated by IRES varied from 6% to 100% compared to the expression of the first gene, depending on cell type and reporter genes, while the efficiency of translation of the second transgene was typically 20–50% lower [22].

Different signal sequences are used for the production of heavy and light chains in the same ratio, which slow down or speed up the production of the chains depending on their properties so that the proper conformation of the antibodies can develop, and neither type of protein chain aggregates [23].

In the present work, our aim was not only to achieve stable transfection and gene expression of Nivolumab-based anti-PD-1 in a mammalian cell line using two expression vector systems but also to optimize the production process. In doing so, we aimed to investigate the effect of different signal sequences on antibody production while proposing a method to increase its overall yield.

## 2. Materials and Methods

### 2.1. Plasmids and Cell Lines

The CHO DG44 cell line used in our study was obtained from UD-GenoMed Medical Genomic Technologies Kft, Debrecen, Hungary. The HC and LC coding sequence of anti-PD-1 was determined based on publicly available information about Nivolumab and codon-optimized expression in CHO DG44 mammalian cells. Codon optimization was achieved using GeneScript’s GenSmart™ Codon Optimization algorithm, which is available online. GeneScript Biotech, Piscataway, NJ, USA, synthesized the LC and HC chains with signal peptide sequences (SPs) of the anti-PD-1 gene (Table 1.).

**Figure 1:**
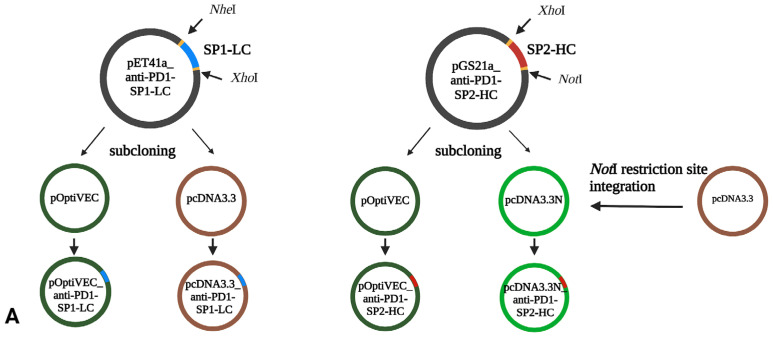

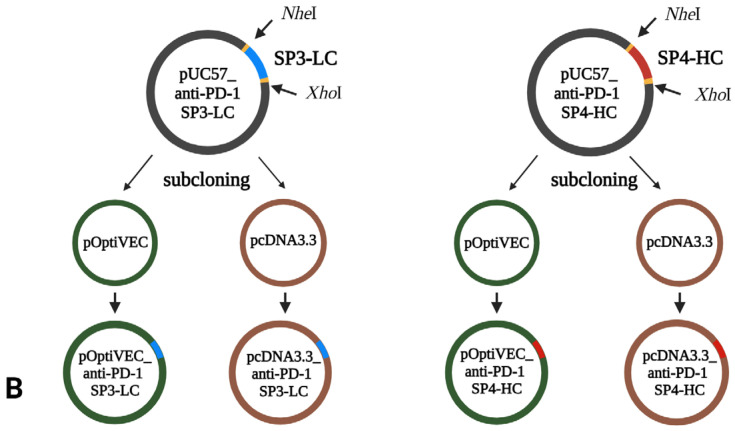
Schematic overview of recombinant plasmid construction (BioRender). (**A**) SP1-LC, SP2-HC; (**B**) SP3-LC, SP4-HC.

The gene for SP1-LC was integrated into the pET41a vector between the recognition sites of *Nhe*I and *Xho*I restriction enzymes. The coding sequence for SP2-HC was integrated into the pGS21a_HC vector between the recognition sites of the restriction enzymes *Xho*I and *Not*I (Figure 1A). The coding sequences for SP3-LC and SP4-HC were integrated into the pUC57 vector with the recognition sites of the restriction enzymes *Nhe*I and *Xho*I (Figure 1B). *E. coli* NEB10-beta, CHO DG44 cells, pOptiVEC, and pcDNA3.3 were generously provided by UD-GenoMed Medical Genomic Technologies, Debrecen. The circular plasmids pOptiVEC and pcDNA3.3 were used as expression vectors for the mammalian cell line CHO DG44.

A 25 bp double-stranded DNA sequence was integrated into the linear vectors pOptiVEC-TOPO and pcDNA3.3-TOPO to generate the circular plasmids pOptiVEC and pcDNA3.3. The integrated sequence introduced an *Nhe*I cleavage site into the vectors.

The primers used in this study were designed using SnapGene^®^ 7.2.1 software (from Dotmatics; available at snapgene.com) and analyzed using Integrated DNA Technologies OligoAnalyzer2.1 (RRID: SCR_001363) software to determine primer–dimer alignment. The primers used to verify the recombinant plasmid were synthesized by UD-Genomed Ltd. The schematic illustration of the recombinant plasmids was created using BioRender software (available online at biorender.com).

### 2.2. Construction of pcDNA3.3N Plasmid

The circular base pcDNA3.3 vector did not contain a *Not*I site suitable for cloning the SP2-HC chain. To construct a *Not*I-containing pcDNA3.3N plasmid, we used the circular vector pcDNA3.3 and modified it by integrating hybridized oligonucleotides to create a NotI restriction site between *Age*I and *Xho*I restriction sites.

Two complementary oligonucleotides of 25 bp were used, with *Age*I and *Xho*I digested sites at the ends, and the *Not*I recognition site was integrated between these restriction sites (Figure 1). The primers were incubated for 10 min at 95 °C in 10 mM TE buffer (10 mM Tris-HCl, pH = 8, 1 mM EDTA) with 50 mM NaCl at a molar ratio of 1:1 in a microcentrifuge tube in a Corbett Research PCR machine and then cooled to 25 °C for 60 min. The oligonucleotides were precipitated with ethanol and stored in a 10 mM Tris-HCl (pH = 8) buffer. The resulting dsDNA was ligated into the pcDNA3.3 plasmid treated with the enzymes XhoI and AgeI (New England Biolabs, NEB, Ipswich, MA, USA) and transformed into chemically competent *E. coli* NEB10. The molar ratio of vector to insert for the ligation reaction was 1:20. The plasmids were isolated from the transformants (GeneJet Plasmid Miniprep Kit, Thermo Fisher Scientific, Waltham, MA, USA) and digested with restriction enzymes for verification (*Not*I–*Pst*I, *Not*I, NEB). The DNA fragments were separated by electrophoresis in a 1% agarose gel (Bio-Rad, Hercules, CA, USA). The structure of the pcDNA3.3N plasmid was verified by DNA sequencing (UD-GenoMed Ltd., Debrecen, Hungary).

### 2.3. Construction of Recombinant Plasmids

To generate the recombinant pcDNA3.3/pOptiVEC_anti-PD1-SP1-LC constructs, the LC sequence from the pET41a_LC plasmid was subcloned into the pcDNA3.3 and pOptiVEC plasmids using the restriction enzymes *Nhe*I/*Xho*I (NEB). To generate recombinant pcDNA3.3N/pOTiVEC_anti-PD1-SP2-HC constructs, the HC sequence from the pGS21a_HC plasmid was subcloned into the pcDNA3.3N and pOptiVEC plasmids using *Xho*I/*Not*I restriction enzymes (NEB). To generate the recombinant pcDNA3.3/pOptiVEC_anti-PD1-SP3-LC constructs, the LC sequence from the pUC57_LC plasmid was subcloned into the pcDNA3.3 and pOptiVEC plasmids using the restriction enzymes *Nhe*I/*Xho*I (NEB). To generate the recombinant pcDNA3.3/pOptiVEC_anti-PD1-SP4-HC constructs, the LC sequence from the pUC57_HC plasmid was subcloned into the pcDNA3.3 and pOptiVEC plasmids using the restriction enzymes *Nhe*I/*Xho*I (NEB).

The ligation products were transformed into chemically competent *E. coli* NEB10. The recombinant plasmids were isolated using the GeneJET Plasmid Miniprep Kit (Thermo Scientific). The structure of the recombinant plasmid was verified using DNA sequencing with primers specific to the vectors. The verified recombinant plasmids were introduced into *E. coli* NEB10 cells using the heat shock method. The plasmids were then isolated using the PureLink™ HiPure Plasmid Midiprep Kit (Thermo Scientific) and linearized using the restriction enzyme PvuI. The linearized plasmids were purified by ethanol precipitation and redissolved in ultrapure water.

### 2.4. Co-Transfection of the CHO DG44 Cells

CHO DG44 cells were cultured in 30 mL CD-DG44 medium (Gibco, Waltham, MA, USA) in 125 mL Erlenmeyer flasks (VWR, Radnor, PA, USA), shaken at 130 rpm on an orbital shaker (PSU-20i, Biosan, Riga, Latvia) in a CO_2_ incubator (HERACell 240, Thermo Scientific) at 37 °C with 8% CO_2_. 48 h before transfection, and the cells were seeded at 1.5 × 10^7^ cells/mL. The transfection mixture contained FreeStyle™ MAX reagent, OptiPRO SFM (Thermo Scientific), and plasmid construct mixture, for which 9 μg of each plasmid construct was used (Table 2). After 48 h, cells were spun at 300 g for 10 min and passaged in OptiCHO (Thermo Scientific) medium supplemented with 8 mM L-glutamine and 500 µL geneticin. The passage of cells was carried out at 95% viability, or a cell count of 10^7^.

### 2.5. Protein Expression

The methotrexate (MTX) amplification was carried out with concentrations of 250 nM, 500 nM, and 1000 nM. Cells were passaged every 3–4 days until cell viability reached 90%. At this point, all MTX-treated cultures were frozen. To test the productivity of the protein, we started the cell cultures with 107 cells and determined the concentration of the produced antibody on the 7th day. The expression of the antibody was verified with enzyme-linked immunosorbent assay (ELISA) using the SHIKARI^®^ Q-NIVO ELISA Kit (Matriks Biotek, Ankara, Türkiye). The SHIKARI^®^ Q-NIVO ELISA Kit operates as a ligand binding assay, where the plate is coated with PD-1, the specific ligand for Nivolumab. This design ensures that only Nivolumab, which binds to PD-1, is quantified. The standards provided in the kit contain the original Nivolumab drug to create a reference curve. The assay was performed in the supernatant according to the manufacturer’s instructions. The optical density was measured at 450 nm using an LEDETECT96 microplate reader (Labexim Products, Lengau, Austria). The quantification was conducted with three biological replicates and at least two independent experiments, ensuring the reliability and reproducibility of the results.

### 2.6. Clonal Selection

The limiting dilution method was used to produce individual clones. The process involves pre-expansion of MTX-amplified cells in CD OptiCHO™ medium, supplemented with 8 mM glutamine, without any selection pressure for a minimum of two passages. On the cloning day, the cells were diluted to a seeding density of 0.5–2 cells per well in a 96-well plate using a specific cloning medium. The medium comprised 86 mL of basal CD FortiCHO™ Medium (Thermo Fisher Scientific, Waltham, MA, USA), 3 mL of freshly thawed 200 mM L-glutamine, 10 mL of conditioned medium, and 1 mL of 100x HT supplement. The cells were serially diluted to a final concentration of 1000 viable cells/mL using a growth medium (CD OptiCHO™ Medium supplemented with 8 mM L-glutamine, Thermo Fisher Scientific, Waltham, MA, USA), resulting in a seeding density of 1 viable cell per well. The cell suspension was gently mixed by inverting the tube 5 or 6 times, and 200 µL of the diluted cells were aseptically dispensed into each of the empty 60 wells of each 96-well plate (the perimeter was filled with sterile PBS solution to avoid evaporation). The plates were then incubated undisturbed for 10–14 days at 37 °C and 5% CO_2_ in humidified air in a static (non-shaking) incubator. The supernatants of individual clones were used for the determination of antibody production using the ELISA assay described before. Two clones with the highest anti-PD-1 concentration were used for further analysis.

### 2.7. Antibody Purification

Protein purification was performed on an FPLC Pharmacia (GE Healthcare Life Sciences, Pittsburg, PA, USA) system using the appropriate columns for each phase. The flow volume was set to 1 mL/min, pressure 0.3 MPa, and protein concentration was measured at 220 nm using a UV detector.

Fractionation was performed on a HiTrap rProtein A HP (Cytiva) 1 mL volume column. The column was pretreated with (Binding Buffer), a 20 mM phosphate buffer, pH = 7. The elution was performed with 100 mM citrate buffer with a pH of 3. The sample volume was 100 µL.

### 2.8. Molecular Weight Determination

With the MALDI-TOF Biotyper (Bruker Daltonics, Bremen, Germany), the molecular weight of the proteins was measured using the 2,5-dihydroxybenzoic acid (DHB) and α-cyano-4-hydroxycinnamic acid (HCCA) matrices, using 0.5 µL matrix + 0.5 µL sample.

The aggregation tendency was determined using a Bio SEC-3 column (Agilent Technologies Inc., Santa Clara, United States) on an HPLC Infinity 1260 system (Agilent Technologies Inc., Santa Clara, United States). The measurements were performed with a flow rate of 1 mL/min, detection at 220 nm UV, and 150 mM phosphate buffer as the eluent.

## 3. Results

### 3.1. Molecular Cloning of Antibodies

To increase the cells’ production capacity, we designed the vectors with two pairs of signal sequences next to the chains based on the literature data. One sequence pair was chosen based on the literature data, and this combination significantly increased the product yield not only in the production of antibodies but also in a wide range of biotechnological products [23].

Since we worked with two vectors and two signal sequence combinations, we assembled the chain separately for HC, LC, and signal sequence combinations by synthesizing both genes with two types of signal sequences and consequently investigating antibody production using different signal peptides.

**Figure 2:**
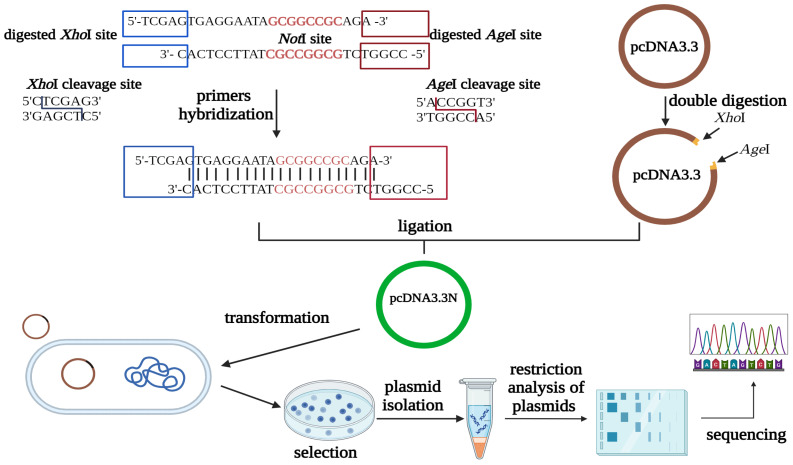
Schematic representation of the pcDNA3.3N plasmid construction (BioRender).

To achieve our research aim, two expression vectors, pOptiVEC and pcDNA3.3, were used for the expression of the antibody in the CHO DG44 cell line. Firstly, we created the pcDNA3.3N plasmid from the circular base vector pcDNA3.3 for cloning anti-PD-1-SP2-HC, into which we integrated the required restriction endonuclease recognition site (*Not*I). The circular base vector pcDNA3.3 did not contain a *Not*I enzyme recognition site suitable for cloning the SP2-HC chain. Therefore, we designed two complementary primers that contain the *Not*I recognition site between the restriction sites *Xho*I and *Age*I. After hybridization of the primers, the resulting dsDNA was ligated into the pcDNA3.3 plasmid pre-treated with *Xho*I and *Age*I enzymes and transformed into a bacterial cloning cell line, as shown in Figure 2.

**Figure 3:**
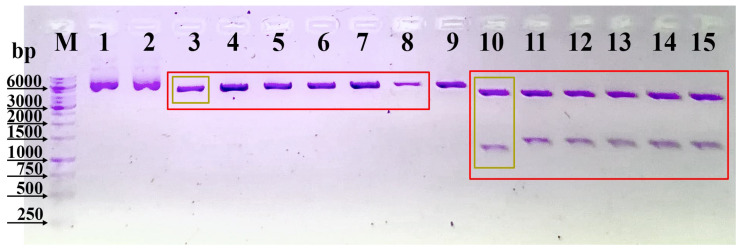
Separation of digested fragments of the pcDNA3.3N plasmid on agarose gel. Lane M. DNA molecular weight marker (1 kb, Thermo Scientific); Lane 1. undigested pcDNA3.3; Lane 2. undigested pcDNA3.3N; Lanes 3–8. pcDNA3.3N digested with *Not*I; Lane 9. pcDNA3.3 digested with *Not*I and *Pst*I; Lanes 10–15. pcDNA3.3N digested with *Not*I and *Pst*I.

Plasmids were isolated from several transformants and digested with various enzymes for verification. The resulting fragments were separated on a 1% agarose gel. In Figure 3, lanes 3–8 show single digests of pcDNA3.3N plasmid (5435 bp), and lanes 10–15 show the results of double digests corresponding to these samples (4077 bp and 1358 bp long products should be obtained). In lane 10, one of the fragments deviates from the expected size, probably indicating improper integration of the DNA target in this case. We selected two transformants from those with the correct sizes and confirmed the construction of the pcDNA3.3N plasmid through sequencing. The sequencing results were compared with the expected sequence using the BioEdit program.

Based on the sequencing data, integrating the *Not*I site into the pcDNA3.3 plasmid successfully created the pcDNA3.3N plasmid.

**Figure 4:**
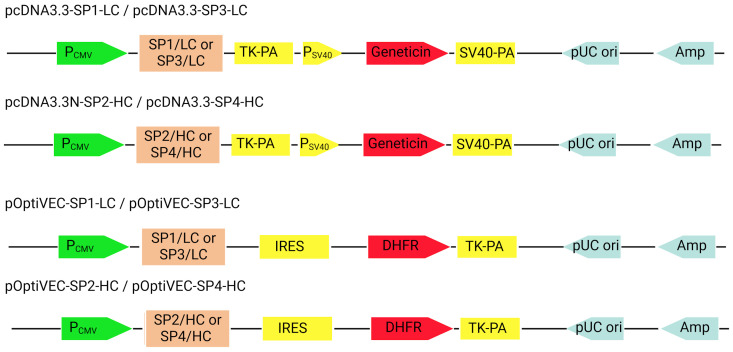
Schematic representation of the recombinant constructs (BioRender). PCMV—cytomegalovirus promoter; TK-PA—thymidine kinase polyA; PSV40—simian virus 40 promoter; geneticin—geneticin resistance gene; IRES—internal ribosome entry site; DHFR—dihydrofolate reductase; Amp—ampicillin resistance gene; pUC ori—bacterial replication origin.

Following successfully constructing the pcDNA3.3N plasmid, we created eight recombinant plasmids (Figure 4) as described in Section 2. The pOptiVEC plasmid contains an IRES site upstream of the DHFR gene, allowing post-transfection selection and amplification (Figure 4). The recombinant plasmids were selected for each type and their correct integration was verified with sequencing. Sequencing was performed in the 5′ and 3′ directions using CMV and IRES primers specific for the pOptiVEC plasmid, and CMV and TK-PA primers specific for the pcDNA3.3 plasmid.

### 3.2. Effect of Signal Peptide Sequences on Antibody Expression

Following confirmation of the correct assembly of the designed expression vectors, the CHO DG44 cells were co-transfected with the four vector combinations, as described in Table 3. After the successful selection, cell pools were treated with different concentrations of MTX for the genomic amplification of the integrated gene, and the production of the antibody was determined with an ELISA test on the 7th day in each case of the different vector combinations. Based on the results presented in Table 3, the highest antibody concentration was found in the case of the sp3/sp4 4 vector combination (pO-SP4-HC/pD-SP3-LC). In the case of 500 nM MTX, we achieved 2.67× higher antibody production than when using 250 nM MTX. No significant difference in antibody production was found on the 7th day when 500 and 1000 nM MTX were used. The best-producing combination proved to be pO-SP4-HC/pD-SP3-LC. The significant difference between antibody titers seems to be the signal sequences.

Monoclones were created from the MTX pool treated with 500 nM using the limiting dilution method, and the antibody concentration was measured using ELISA. A number of seven of the clones tested showed significant antibody production, with the highest antibody levels obtained being 74.5 mg/L.

### 3.3. Determination of the Molecular Weight

**Figure 5:**
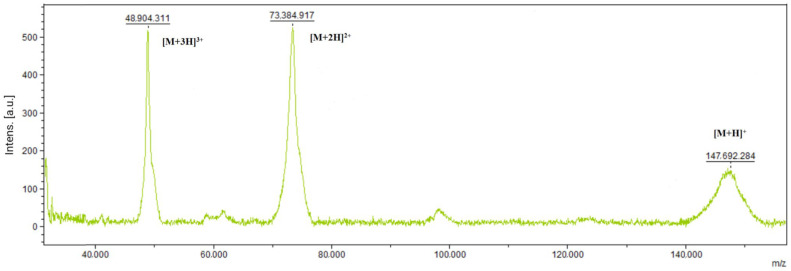
Determination of the molecular weight of Nivolumab with MALDI-TOF. The peak appearing at 146 kDa is Nivolumab. Ionization fragments are visible at 48 kDa and 73 kDa, respectively.

The purified proteins’ molecular weights were determined using mass spectrometry on a MALDI-TOF Biotyper. Figure 5 shows the molecular weight measured with an α-cyano-4-hydroxycinnamic acid (HCCA) matrix. Nivolumab’s molecular weight is 146 kDa, so the obtained size meets expectations.

### 3.4. Determination of Aggregation Tendency by HPLC on a SEC-3 Column

**Figure 6:**
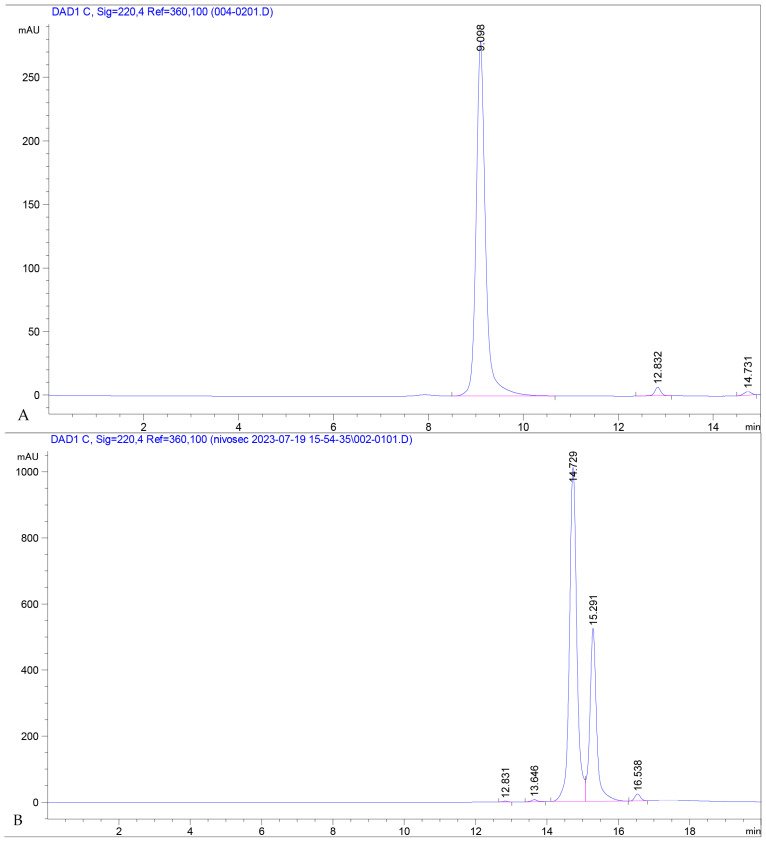
The (**A**) chromatogram shows fractions of the intact, purified antibody. The (**B**) chromatogram represents a sample reduced with 1 mM GSH and separated under the same conditions. The first large peak at an RT of 14.729 represents the heavy chain, the next one, at 15.291 is the light chain, and the third small peak represents the non-glycosylated heavy chain.

The size, type, and content of aggregates in protein biopharmaceuticals can influence efficacy and induce an immunogenic response. Aggregation formations occur through several mechanisms, including disulfide bond formation and non-covalent interactions. Since the size of protein aggregates, including dimers, is sufficiently different from the protein monomer, it is possible to separate the variable forms using SEC (size exclusion chromatography). SEC measured under UV light is a standard technique for quantitatively determining protein aggregation and molecular weight determination. In Figure 6, we can see the intact and separated forms of the produced antibody under reducing conditions. In its intact form, 97.64% is in the correct monomeric conformation, meeting FDA and EMA requirements. A total of 64.67% of the fragments of reduced antibodies are heavy chains, 33.89% are light chains, and 0.97% are non-glycosylated heavy chains.

## 4. Discussion

Recombinant biotherapeutic proteins, such as monoclonal antibodies, are produced prominently in CHO cells, for which high-producing scalable platform development is still a valid objective. In addition to vector optimization with transcription and translation level regulatory elements, signal peptide selection significantly affects antibody productivity and secretion efficiency. Several works trigger the enhancement of antibody production by signal sequence optimization, both in CHO and other cell lines [24,25]. Some works report higher antibody titers obtained by using natural signal peptides from human serum albumin and azurocidin [23] or report the optimization of sequences derived from human immunoglobulin (Ig) heavy chain (HC) and kappa light chain (LC) [26].

During our research, we developed a co-expression vector system for the stable expression of anti-PD-1 in a mammalian cell line. We created combinations of different signal sequences for increased antibody titers. For this purpose, we constructed the plasmid pcDNA3.3N starting from pcDNA3.3 and rendered it suitable for cloning the HC of Nivolumab by integrating the *Not*I restriction recognition site. We then successfully generated eight recombinant plasmids. The structure of the plasmid constructs was verified with DNA sequencing.

Co-transfection of CHO DG44 cells was carried out with different combinations of recombinant vectors (pO-SP1-LC/pD-SP2-HC, pO-SP2-HC/pD-SP1-LC, pO-SP3-LC/pD-SP4-HC, pO-SP4-HC/pD-SP3-LC), and a stable cell pool was subsequently generated.

Monoclones were created from the stable pools by limiting dilution, and then antibody production was measured using the ELISA method. The best-producing clones were created by the vector combination pO-SP4-HC/pD-SP3-LC. Antibodies from the best-producing monoclonal were purified and analyzed to demonstrate protein similarity on the first level.

## 5. Conclusions and Future Perspectives

In conclusion, our study successfully developed a stable expression system for producing anti-PD-1 antibodies using CHO DG44 cells. Using different co-expression vectors and signal peptides, we identified a combination that significantly improved antibody titers, particularly with the pO-SP4-HC/pD-SP3-LC combination. These findings underline the importance of signal peptide selection in optimizing monoclonal antibody production for therapeutic use.

We aim to optimize production conditions and scale up processes further to meet industrial standards. Additionally, we plan to perform detailed analyses, including peptide mapping and glycosylation profiling, to ensure the quality and functionality of the produced antibodies. In perspective, we will conduct in vitro functional assays, such as PD-1/PD-L1 blockade bioassays and T-cell cytotoxicity assays, to confirm the bioactivity of the antibodies. These future efforts could enhance the commercial viability of this production method and contribute to advancements in biopharmaceutical manufacturing.

## Figures and Tables

**Table 1:** The most important data of the paired signal peptide sequences.

Name of Construct	Signal Peptide Sequence	GenBank Accession Number	Protein
SP1-LC	MKWVTFISLLFLFSSAYS	NP_000468 *	Serum albumin preproprotein
SP2-HC	MTRLTVLALLAGLLASSRA	NP_001691 *	Azurocidin preproprotein
SP3-LC	METPAQLLFLLLWLPDTTG	P18136 **	Ig kappa chain V-III region HIC
SP4-HC	MEFGLSWLFLVAILKGVQC	P01764 **	Ig heavy chain V-III region VH26

* NCBI database, ** Uniprot database.

**Table 2:** Characteristics of the recombinant construct combinations used in this study.

Short Name of the Vector Combination	Vector Combination	Signal Peptide Combination
pO-SP1-LC/pD-SP2-HC	pOptiVEC_anti-PD1-SP1-LC and pcDNA3.3N_anti-PD1-SP2-HC	SP1-LC and SP2-HC
pO-SP2-HC/pD-SP1-LC	pOptiVEC_anti-PD1-SP2-HC and pcDNA3.3_anti-PD1-SP1-LC	SP1-LC and SP2-HC
pO-SP3-LC/pD-SP4-HC	pOptiVEC_anti-PD1-SP3-LC and pcDNA3.3_anti-PD1-SP4-HC	SP3-LC and SP4-HC
pO-SP4-HC/pD-SP3-LC	pOptiVEC_anti-PD1-SP4-HC and pcDNA3.3_anti-PD1-SP3-LC	SP3-LC and SP4/HC

**Table 3:** Antibody titers (mean +/− SD) of selected polyclones from the different vector/signal sequence combinations tested after 7 days of MTX treatment.

Vector Combination	Antibody Concentration, mg/L		
	250 nM MTX	500 nM MTX	1000 nM MTX
pO-SP1-LC/pD-SP2-HC	3.12 ± 0.18	7.29 ± 0.15	5.78 ± 0.12
pO-SP2-HC/pD-SP1-LC	3.93 ± 0.11	20.13 ± 0.13	21.30 ± 0.20
pO-SP3-LC/pD-SP4-HC	4.78 ± 0.09	12.94 ± 0.18	21.55 ± 0.23
pO-SP4-HC/pD-SP3-LC	22.82 ± 0.12	60.93 ± 0.1	60.21 ± 0.13

## Data Availability

Data are contained within the article.
